# Temporal and mechanistic dissociation of ATP and adenosine release during ischaemia in the mammalian hippocampus^1^

**DOI:** 10.1111/j.1471-4159.2006.04425.x

**Published:** 2007-06

**Authors:** Bruno G Frenguelli, Geoffrey Wigmore, Enrique Llaudet, Nicholas Dale

**Affiliations:** * Neurosciences Institute, Division of Pathology & Neuroscience, University of Dundee, Ninewells Hospital Dundee, UK; † Department of Biological Sciences, University of Warwick Coventry, UK

**Keywords:** ATP, adenosine, ischaemia, hypoxia, purines, peri-infarct depolarisation

## Abstract

Adenosine is well known to be released during cerebral metabolic stress and is believed to be neuroprotective. ATP release under similar circumstances has been much less studied. We have now used biosensors to measure and compare in real time the release of ATP and adenosine during *in vitro* ischaemia in hippocampal slices. ATP release only occurred following the anoxic depolarisation, whereas adenosine release was apparent almost immediately after the onset of ischaemia. ATP release required extracellular Ca^2+^. By contrast adenosine release was enhanced by removal of extracellular Ca^2+^, whilst TTX had no effect on either ATP release or adenosine release. Blockade of ionotropic glutamate receptors substantially enhanced ATP release, but had only a modest effect on adenosine release. Carbenoxolone, an inhibitor of gap junction hemichannels, also greatly enhanced ischaemic ATP release, but had little effect on adenosine release. The ecto-ATPase inhibitor ARL 67156, whilst modestly enhancing the ATP signal detected during ischaemia, had no effect on adenosine release. Adenosine release during ischaemia was reduced by pre-treament with homosysteine thiolactone suggesting an intracellular origin. Adenosine transport inhibitors did not inhibit adenosine release, but instead they caused a twofold increase of release. Our data suggest that ATP and adenosine release during ischaemia are for the most part independent processes with distinct underlying mechanisms. These two purines will consequently confer temporally distinct influences on neuronal and glial function in the ischaemic brain.

The purines ATP and adenosine exert powerful modulatory influences in the mammalian central nervous system. ATP can act at both ligand-gated ATP P2X receptors and G protein-coupled P2Y receptors, whereas adenosine acts only through four G protein-coupled receptors (Ralevic and Burnstock 1998). Purinergic signalling influences transmitter release (Dunwiddie and Masino 2001), synaptic plasticity (Pankratov *et al.* 2002; Pascual *et al.* 2005), neurone-glia interactions (Fields and Burnstock 2006), nociception (Liu and Salter 2005), sleep-wake cycles (Basheer *et al.* 2004), respiratory (Gourine 2005) and locomotor rhythms (Dale and Kuenzi 1997), anxiety, depression, aggression and addiction (Fredholm *et al.* 2005).

Adenosine is well known to be released during cerebral hypoxia/ischaemia both *in vitro* and *in vivo* (Latini and Pedata 2001; Frenguelli *et al.* 2003; Phillis and O’Regan 2003). Indirect studies using pharmacological antagonists (Fowler 1989; Pearson *et al.* 2006), receptor knockouts (Johansson *et al.* 2001) or focal receptor deletion (Arrigoni *et al.* 2005) demonstrate that activation of presynaptic adenosine A_1_ receptors causes rapid depression of excitatory synaptic transmission during hypoxia/ischaemia *in vitro* and *in vivo* (Gervitz *et al.* 2001; Ilie *et al.* 2006). This conclusion is strengthened by the close temporal association of adenosine release with the depression of excitatory synaptic transmission (Frenguelli *et al.* 2003; Pearson *et al.* 2006). Activation of A_1_ receptors is widely regarded as an important aspect in the neuroprotection provided by adenosine (Sebastiao *et al.* 2001; Arrigoni *et al.* 2005).

Intracellular ATP falls dramatically during cerebral metabolic stress *in vitro* (Gadalla *et al.* 2004) and *in vivo* (Phillis *et al.* 1996). The issue of whether ATP, like adenosine, is also released during cerebral ischaemia has not been extensively examined. Direct release of ATP has been demonstrated *in vitro* (Juranyi *et al.* 1999) and *in vivo* (Melani *et al.* 2005), but these HPLC studies lack good spatial and temporal resolution. In contrast, some studies have failed to demonstrate ATP release (Phillis *et al.* 1993). Indirect evidence, such as extracellular metabolism of nucleotides to adenosine (Koos *et al.* 1997) or the post-ischaemic up-regulation of ATP metabolising ectoenzymes (Braun *et al.* 1998) is suggestive of ATP released during metabolic stress. However, unlike adenosine release, the timing, dynamics and quantity of ATP release during ischaemia has not been documented.

In this paper, we have used enzyme-based microelectrode biosensors (Frenguelli *et al.* 2003; Dale *et al.* 2005; Llaudet *et al.* 2005) to measure simultaneously the real-time release of adenosine and ATP during ischaemia in rat hippocampal slices. This has allowed us to study in detail the quantity, timing and mechanisms of ATP release. We find that ATP is released only following the anoxic depolarisation, well after the initial release of adenosine. Relatively small quantities of ATP are released compared with adenosine and the mechanisms of ATP and adenosine release are quite distinct.

## Methods

### Electrophysiology

Extracellular recordings were made from area CA1 of 400 μm hippocampal slices from 11–16 and 22–27 days old Sprague-Dawley rat pups. Slices, prepared as described previously (Dale *et al.* 2000), were suspended on a mesh and submerged in aCSF flowing at 5–6 mL/min at 33–34°C. Field excitatory postsynaptic potentials (fEPSPs) were recorded, with aCSF-filled glass microelectrodes, from stratum radiatum of area CA1 in response to stimulation (at 15 s intervals; bipolar Teflon-coated tungsten wire) of the Schaffer collateral-commissural fiber pathway. ‘Blind’ whole-cell patch clamp recordings were made in current-clamp mode from CA1 pyramidal neurones using pipettes (5–7 MΩ) containing (in mmol/L): K-gluconate, 130; KCl, 10; CaCl_2_, 2; EGTA, 10; HEPES, 10; pH 7.27, adjusted to 295 mOsm.

Standard aCSF contained (in mmol/L): NaCl, 124; KCl, 3; CaCl_2_, 2; NaHCO_3_, 26; NaH_2_PO_4_, 1.25; d-glucose, 10; MgSO_4_, 1; pH 7.4 with 95% O_2_/5% CO_2_ and was gassed with 95% O_2_/5% CO_2_. In ‘ischaemic’ aCSF, 10 mmol/L sucrose replaced the 10 mmol/L d-glucose and was equilibrated with 95% N_2_/5% CO_2_ (Frenguelli 1997; Pearson *et al.* 2006). As previously reported, (Dale *et al.* 2000), this substitution of N_2_ for O_2_ caused a rapid decrease in the bath oxygen tension from approximately 80–90% saturation to <10%. The recording and analysis of fEPSPs was under the control of LTP software (courtesy of Dr Bill Anderson & Professor Graham Collingridge, University of Bristol, http://www.ltp-program.com/indexLtp24.htm (Anderson and Collingridge 2001)). For the most part, electrical recordings were filtered between 1 Hz and 3 kHz (sampling frequency 10 kHz), but to record accurately the extracellular potential shifts associated with the anoxic depolarisation, continuous DC − 3 kHz recordings were also made using the custom software package used to record sensor signals (see below).

### ATP and adenosine sensors

The principles and operation of the Pt/Ir microelectrode biosensors for the purines, ATP and adenosine, have been described previously (Frenguelli *et al.* 2003; Llaudet *et al.* 2003, 2005). These biosensors have been used to record: release of ATP from spinal cord during locomotion (Llaudet *et al.* 2005); release of ATP in the developing retina (Pearson *et al.* 2005) and release of ATP from the ventral medulla oblongata during hypercapnia (Gourine *et al.* 2005a) and hypoxia (Gourine *et al.* 2005b). The biosensors in this study were obtained from Sarissa Biomedical (http://www.sarissa-biomedical.com; Coventry, UK) and were a development of those previously described in that the Pt/Ir microelectrode was coated with a permselective polymer prior to enzyme matrix deposition. This ‘screening layer’ greatly improves the selectivity of the biosensors by reducing any contribution to the signal by electroactive interferents such as ascorbate, 5-HT or noradrenaline. Before insertion and after removal of the sensors from the slice, the sensors were calibrated with 10 μmol/L ATP, adenosine and 5-HT, the latter being used to assess the patency of the screening layer.

To give a measure of net ATP concentrations the signal from a ‘Null’ sensor (lacking enzymes, but otherwise identical) was used to measure background signals, which were then subtracted from the signals generated by the ATP biosensor. For the adenosine sensor, a separate ‘inosine’ sensor, lacking adenosine deaminase, is required to yield a signal specific to adenosine (Frenguelli *et al.* 2003). To limit the number of sensors inserted into the slice, this additional sensor was largely omitted. Thus, the signal recorded from the adenosine sensor can be described as that from both adenosine and inosine (ado/ino). From previous studies, we have estimated that approximately 50% of the signal from the ado/ino biosensor is due to inosine (Dale *et al.* 2000; Frenguelli *et al.* 2003). To take into account that biosensor sensitivity varies during different experiments, we have used the calibration of the sensor to adenosine to normalise the signal. We report the values in μmol/L′ to indicate that the signal is normalised to sensitivity but cannot be literally interpreted in units of concentration for adenosine or inosine but is a mixture of the two substances. The ado/ino signal displays a negative-going deflection during the onset of ischaemia that reflects a slight sensitivity to O_2_ sensitivity of the background current of the sensor, and not a decrease in extracellular adenosine (Frenguelli *et al.* 2003).

ATP, Null and ado/ino biosensors were each inserted through the 400 μm thickness of the slice such that most of the sensing part of the sensors was in intimate contact with the tissue. We have previously shown that this is not detrimental to the slice and that fEPSPs can be recorded with the sensors in place, and indeed directly from the sensors (Frenguelli *et al.* 2003). Additionally, the stimulating and recording electrodes were placed adjacent to the sensors. Continuous signals from the sensors and recording electrode were acquired at 10 kHz using a custom software package and displayed on a PC. The software allowed *post-hoc* compensation for any differences in sensor sensitivity and subtractions to be performed to yield net ATP signals, which were expressed in absolute units of concentration (μmol/L).

### Experimental protocols

Drugs were bath applied to the slice and sufficient time, at least 15 min, was allowed for their equilibration through the tissue. Drugs were either applied as aliquots from stock solutions or dissolved directly into the aCSF (kynurenic acid, carbenoxolone, EGTA, homocysteine thiolactone). Ca^2+^-free aCSF comprised aCSF to which no Ca^2+^ was added, but contained an extra 2 mmol/L Mg^2+^ to maintain the concentration of divalent cations. To further reduce extracellular Ca^2+^, 1 mmol/L EGTA was added to this nominally Ca^2+^-free aCSF.

### Malachite Green phosphate assay and measurement of ectonucleotidase activity in slices

To assess endogenous ectonucleotidases activity in hippocampal slices and the efficacy of ARL 67156 as a blocker, we used the convenient phosphate assay of Carter and Karl (1982).

Hippocampal slices (400 μm thickness) were prepared from 24–26 days male Sprague-Dawley rats. They were carefully dissected free from the cortex, thalamus and globus pallidus and kept in oxygenated aCSF solution at room temperature for 1 h prior to being placed into individual wells in a 24 well plate. The hippocampal slices were then washed five times with phosphate-free aCSF (comprising (in mmol/L): KCl, 3.1; NaCl, 126; NaHCO_3_, 26; d-glucose, 10; CaCl_2_, 1; MgCl_2_, 2).

The slices (one per well) were then immersed in 400 μL of test solution (phosphate-free aCSF with either 10, 50 or 100 μmol/L ATP). 5% CO_2_ and 95% O_2_ was blown across the surface of each well to prevent hypoxia. The multi-well plate was placed on a shaker so that there was gentle agitation of the slices and good mixing within the wells. To maintain the health of the slices each experimental run was restricted to 30 min, with 25 μL aliquots being removed at 0, 1, 3, 5, 10, 15, 20, 25 and 30 min for phosphate analysis.

The rate of phosphate production (measured from the slope of the graph of [phosphate] versus time) was compared in the control to the rate in the presence of ARL 67156. A timed control was also performed whereby the rate of phosphate production was compared for two successive runs without the addition of ARL 67156. This resulted in a small reduction of the rate of phosphate production between runs of 9.8 ± 3.3% (*n* = 16). This small decrease in the control was subtracted from the decreases caused by ARL 67156 to give the net reduction in phosphate production due to the inhibition of the ectoATPases.

### Drugs

The salts used in the aCSF were obtained from Fischer. EGTA, kynurenic acid, carbenoxolone, ouabain, S-(4-Nitrobenzyl)-6-thioinosine (NBTI), dipyridamole (DIPY), *L*-homocysteine thiolactone, adenosine, inosine and 5-HT were from Sigma–Aldrich. TTX was purchased from Alomone Labs, ARL 67156 from Tocris and ATP from Boehringer.

### Data analysis and statistics

Data are expressed as mean ± SEM and were pooled and compared using unpaired *t*-tests. Computed significance values are given where appropriate.

## Results

### Estimates of basal adenosine and ATP concentrations in area CA1 of hippocampal slices

The selectivity of the biosensors versus common interferences found in the brain makes it possible to estimate the basal tone of adenosine and ATP in the slice. As the electrochemical sensors have a background current this determination can most conveniently be performed via a two-stage method, by first inserting the sensor into the slice, allowing it to equilibrate for at least 30 min and then measuring the change in current observed when the sensor is pulled from the slice. To convert this change in current to absolute concentration of adenosine or ATP we used an inosine and null sensors, respectively, as references for the adenosine and ATP measurements.

In slices that had not been subjected to ischaemia, there was no detectable ATP tone (*n* = 4). Similarly, in 3/4 slices no detectable adenosine tone could be measured (although a tone of 210 nmol/L was measured in one slice). The inability to consistently detect the adenosine tone, readily observable on excitatory synaptic transmission through the use of adenosine A_1_ antagonists is possibly because of the limitations of the two-stage measuring method, as pharmacological manipulations to reduce the breakdown of ATP consistently revealed a loss of adenosine tone (see later). However, in slices that had been subjected to ischaemia, when measured some 30 min following the end of the ischaemic episode, an adenosine tone was detectable (mean 340 ± 130 nmol/L, *n* = 6; see also Pearson *et al.* 2006). An ATP tone (2.5 and 0.1 μmol/L) was measured in 2/6 slices that had been exposed to ischaemia.

### Differential release of adenosine and ATP during ischaemia

To mimic the oxygen/glucose deprivation that occurs during *in vivo* ischaemia, we switched the superfusate of hippocampal slices from a control aCSF to an ‘ischaemic’ aCSF that had been pre-equilibrated with N_2_ and lacked d-glucose. The mean duration of the ischaemic episodes given under the various experimental conditions is provided in Table S1.

**Fig. 1:**
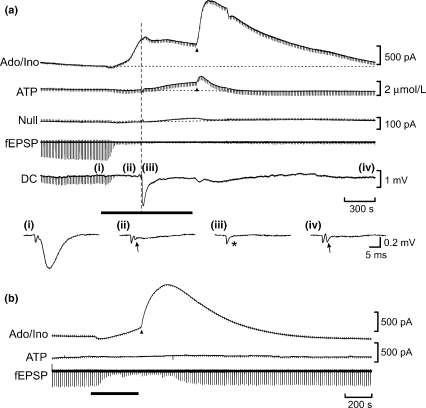
Real-time measurement of the release of ATP and adenosine during ischaemia *in vitro*. (a) Simultaneous recordings from adenosine/inosine (Ado/ino), ATP and null biosensors, the fEPSP (in AC mode) and extracellular DC potential. Ischaemia (black bar) caused the rapid depression of the fEPSP (disappearance of the periodic negative deflections on the traces), which was associated with a rise in extracellular adenosine. Little change is seen in the ATP signal in the early stages of ischaemia. As the ischaemic episode progressed the anoxic depolarisation occurred (large negative deflection on DC trace; dashed vertical line). ATP release followed the anoxic depolarisation. Re-oxygenation resulted in a characteristic surge of adenosine release (the post hypoxic/ischaemic efflux; PPE; arrowhead) and, to a lesser extent, of ATP (arrowhead). Synaptic transmission did not recover after the ischaemic episode in this slice. Magnified examples of the fEPSP shown at the times indicated on the continuous DC trace are shown. The arrow indicates the fibre volley. Note that this has disappeared in trace (iii) (asterisk), obtained shortly after the anoxic depolarisation. On re-oxygenation, the fibre volley was usually enhanced (iv). (b) A short period of ischaemia (black bar), insufficient to evoke the anoxic depolarisation, did not elicit ATP release, but a substantial amount of adenosine was released both during and after the ischaemic episode. Note that full recovery of the fEPSP occurred. The rapid periodic deflections on the biosensor traces are a combination of fEPSP and artefacts from the stimulating electrode used to evoke the fEPSP. The negative deflection on the Ado/ino trace arises from removal of oxygen and does not represent a fall in extracellular adenosine/inosine concentration. In this and subsequent figures the ATP sensor trace reflects net ATP, i.e. the signal after subtraction of the simultaneously-recorded null sensor.

As previously described (Frenguelli 1997; Pearson *et al.* 2006), this resulted in a rapid depression of excitatory synaptic transmission (<10% of control after 3 min of ischaemia, *n* = 13). The ado/ino biosensor exhibited a rapidly increasing current, which reached 338 ± 36 pA (*n* = 18) at 5 min (Figs 1a and b; cf. Frenguelli *et al.* 2003). By contrast, no change in current was recorded on either the ATP or Null biosensors at 5 min (*n* = 19; Figs. 1a and b). Thus, the signal recorded by the ado/ino biosensor represented a specific purine nucleoside signal and was equivalent to 3.3 ± 0.4 μmol/L′ (Table 1). This confirms previous observations of the dominant role of adenosine in the hypoxic/ischaemic depression of glutamatergic transmission (Fowler 1989; Johansson *et al.* 2001; Arrigoni *et al.* 2005; Pearson *et al.* 2006). Our observation of ado/ino release in the apparent absence of ATP release during ischaemia suggests that the majority of extracellular adenosine does not arise from the prior release and degradation of ATP.

**Table 1:** Characteristics of ATP and adenosine release in response to *in vitro* ischaemia

	Net ATP (μmol/L)			(μmol/L s)		Adenosine (μmol/L′)				(mmol/L′ s)	
Condition	AD	ReOx	PPE	Integral	n	5 min	AD	ReOx	PPE	Integral	*n*
control	0.1 ± 0.1	0.7 ± 0.2	1.8 ± 0.2	897 ± 133	19	3.3 ± 0.4	8.9 ± 0.8	9.2 ± 0.7	22.7 ± 1.6	12.0 ± 1.0	18
Ca^2+^-free	0.2 ± 0.1	0.8 ± 0.2	1.2 ± 0.3	756 ± 174	5	10.0 ± 1.2 *p* = 1.6 × 10^−6^	10.9 ± 1.3	8.4 ± 1.3	15.9 ± 2.4 *p* = 0.051	9.4 ± 0.9	5
Ca^2+^-free & EGTA	−0.2 ± 0.1	−0.1 ± 0.2	0.4 ± 0.1	85 ± 77 *p* = 0.004	5	13.7 ± 1.9 *p* = 5 × 10^−8^	17.1 ± 2.5	9.3 ± 2.7	17.6 ± 2.3	14.1 ± 3.6	6
Kynurenate	0.4 ± 0.2	1.4 ± 0.5	3.7 ± 0.9 *p* = 0.0064	1317 ± 303	6	2.7 ± 0.6	8.2 ± 1.8	6.1 ± 1.4	18.3 ± 3.7	11.5 ± 3.0	5
TTX	0.2 ± 0.2	1.4 ± 0.2	3.7 ± 0.7 *p* = 0.0019	1224 ± 430	4	2.6 ± 0.5	9.0 ± 1.2	10.9 ± 1.4	21.7 ± 4.5	12.8 ± 2.8	4
Carbenoxolone	0.3 ± 0.2	2.9 ± 1.1	4.8 ± 1.2 *p* = 0.0006	2954 ± 369 *p* = 2 × 10^−6^	5	6.3 ± 2.4	8.2 ± 2.3	12.2 ± 2.1	35.6 ± 10.4 *p* = 0.041	13.4 ± 2.4	5
ARL	0.3 ± 0.1	1.1 ± 0.3	3.3 ± 0.7 *p* = 0.017	1369 ± 317	7	2.7 ± 0.3	7.6 ± 1.0	9.0 ± 1.3	20.7 ± 1.9	10.6 ± 1.7	7
Homocysteine thiolactone	1.5 ± 0.6	1.9 ± 0.9	3.0 ± 0.8 *p* = 0.058	1930 ± 672 *p* = 0.024	5	2.4 ± 0.7	7.3 ± 1.2	7.6 ± 1.0	13.8 ± 2.6 *p* = 0.014	8.2 ± 1.8	5
DIPY/NBTI	0.2 ± 0.1	0.7 ± 0.2	1.6 ± 0.4	911 ± 168	4	5.0 ± 1.1	19.4 ± 3.9	13.1 ± 1.5	33.2 ± 5.9 *p* = 0.022	24.0 ± 6.3 *p* = 0.0028	4

Measurements of net ATP and adenosine release were made at the time of the anoxic depolarisation (AD), at the time of re-oxygenation (ReOx), at the peak of the post ischaemic purine efflux (PPE). See Figure S1 for annotation of a record showing where the measurements were made. Additionally, a value for adenosine release at 5 min of ischaemia is given (5 min). Note that at the time of the anoxic depolarisation, there was no significant release of ATP – this occurred progressively following the anoxic depolarisation (reflected in the significant values seen at the time of re-oxygenation). The integral was calculated as the area under the curve from the onset of release until 30 min after the end of the ischaemic episode or until the return of the purine signal to baseline. Data are expressed as mean ± SEM; *n* refers to the number of slices. Where probability values are given they were calculated from a two sample *t*-test comparing the test value to the equivalent control value.

As the period of ischaemia was prolonged (Fig. 1a), the neurons in the slice went through a phenomenon termed the ‘anoxic depolarisation’ or ‘hypoxic spreading depression’ (Somjen 2001). The anoxic depolarisation was detected as loss of the presynaptic fiber volley rapidly followed by a large negative deflection on the extracellular DC trace. Ado/ino release continued to increase to the point of the anoxic depolarisation reaching 8.9 ± 0.8 μmol/L′. However, ATP was only released following the anoxic depolarisation, reaching a concentration of 0.7 ± 0.2 μmol/L at the point of re-oxygenation (usually 2–4 min following the anoxic depolarisation). By contrast ado/ino release either reached a plateau following the anoxic depolarisation or began to decline (overall the signal was 9.2 ± 0.7 μmol/L′ at the moment of re-oxygenation).

Following re-oxygenation we observed a surge in ado/ino release (previously reported as the post-hypoxic purine efflux, PPE (Frenguelli *et al.* 2003)), which reached a peak of 22.7 ± 1.6 μmol/L′ (Fig. 1a, Table 1). A much smaller and shorter-lasting version of the PPE was observed on the ATP biosensor signal that reached a peak of 1.8 ± 0.2 μmol/L and rapidly fell back to baseline. By contrast with the ado/ino and ATP biosensor signals, the Null sensors recorded only small currents at the point of the anoxic depolarisation (20 ± 4.7 pA; *n* = 19; Fig. 1a) and at the moment of re-oxygenation (63 ± 10 pA), with no evidence of a surge in current following re-oxygenation. The signals recorded by the ATP and ado/ino sensors are thus most likely the result of their specific analyte rather than a non-specific interferent.

Shorter episodes of ischaemia (Fig. 1b, *n* = 4), which did not progress to the anoxic depolarisation, confirmed that ado/ino release can be observed in the apparent absence of any ATP release – no ATP release was seen either during ischaemia or following re-oxygenation, in stark contrast to the large ado/ino signal at these time points.

Interestingly, we found that ATP release during ischaemia was sensitive to age. In studies where we made a direct comparison, the peak of ATP release following re-oxygenation was 0.8 ± 0.3 μmol/L (*n* = 6) in slices derived from 11–15 days rats and 2.4 ± 0.8 μmol/L (*n* = 8) in slices derived from 22–27 days rats (*p* = 0.069, one-sided *t*-test; data not shown). In these experiments, the anoxic depolarisation and ATP release occurred later in the younger animals (14.2 ± 1.6 min; *n* = 4) compared with the older group (7.9 ± 0.7 min; *n* = 4; *p* = 0.007).

The association between the anoxic depolarisation and ATP release was further strengthened by the use of ouabain, an inhibitor of the Na^+^/K^+^-ATPase, which mimics the anoxic depolarisation (Tanaka *et al.* 1997) and is used to elicit anoxic depolarisation-like events in brain tissue (Joshi and Andrew 2001). Oubain (100 μmol/L; *n* = 2; data not shown) rapidly elicited an anoxic depolarisation-like negative shift on the DC trace and simultaneously caused an increase in both extracellular ado/ino and ATP.

### Whole cell recordings confirm the relationship between anoxic depolarisation and ATP release

**Fig. 2:**
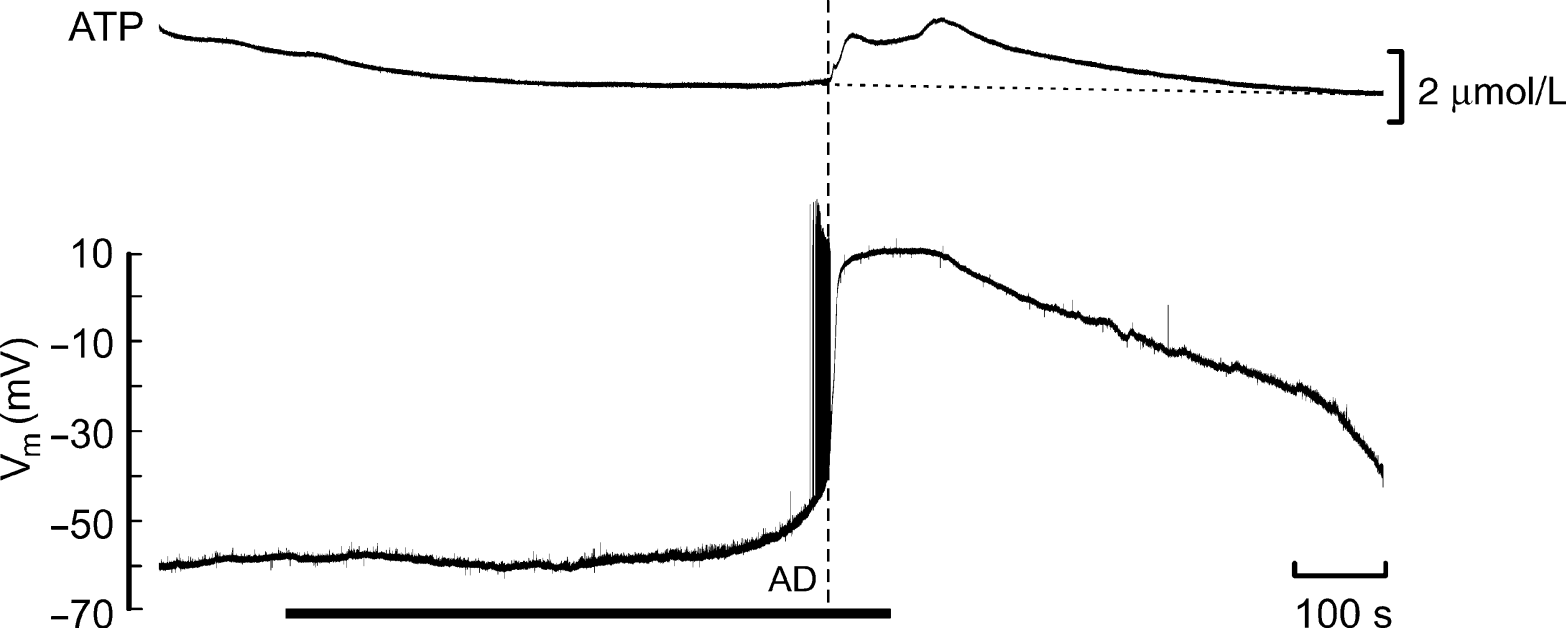
Close temporal association between ATP release and the anoxic depolarisation. Simultaneous extracellular ATP measurement (upper trace) and whole-cell current-clamp recording of membrane potential in a CA1 pyramidal neurone (lower trace) during ischaemia (black bar). Following a gradual depolarisation, during which there was no change in the signal on the ATP sensor, a rapid membrane depolarisation (the anoxic depolarisation, dashed vertical line) occurred provoking repeated action potential firing. ATP release occurred immediately after the anoxic depolarisation.

To examine the timing of ATP release with respect to the anoxic depolarisation in more detail, we performed whole-cell patch-clamp recordings from CA1 pyramidal neurons during ischaemia (Fig. 2). These current-clamp recordings (*n* = 10) revealed that as the ischaemic episode progressed, the neurons underwent a slow period of depolarisation that abruptly increased at the point of the anoxic depolarisation. ATP release only occurred following this rapid depolarisation of the neurons.

### Differential sensitivity of ATP and adenosine release to extracellular Ca^2+^

As ATP can be released in a Ca^2+^-dependent manner (Sperlágh and Vizi 1996; Bodin and Burnstock 2001; Pankratov *et al.* 2006), we asked whether the release of ATP during ischaemic conditions was Ca^2+^-dependent.

**Fig. 3:**
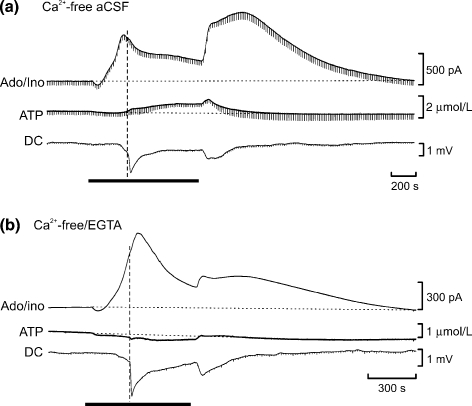
Removal of extracellular Ca^2+^ reveals mechanistic differences between ischaemic ATP and adenosine release. (a) Removal of Ca^2+^ from the aCSF and substitution by Mg^2+^ abolished synaptic transmission, accelerated the anoxic depolarisation (dashed vertical line), enhanced the early release of adenosine during ischaemia (black bar), but did not alter ATP release. (b) In contrast, substitution and chelation of extracellular Ca^2+^ with 2 mmol/L Mg^2+^/1 mmol/L EGTA, respectively, eliminated ATP release caused by the anoxic depolarisation and greatly reduced the post-ischaemic efflux of ATP.

As an initial test of the Ca^2+^-dependency of purine release during ischaemia we omitted extracellular Ca^2+^ and added an extra 2 mmol/L Mg^2+^ to maintain the total concentration of divalent cations. This modified aCSF was perfused onto hippocampal slices (Fig. 3a). As expected, this substitution caused a complete inhibition of the fEPSP, but also had the effect of accelerating both the loss of the presynaptic fibre volley and the anoxic depolarisation (time from onset of ischaemia to the anoxic depolarisation was 459 ± 18 s in control and 315 ± 6 s in zero Ca^2+^ aCSF (Somjen 2001)). In these nominally Ca^2+^-free conditions, adenosine release still occurred and indeed was greater than in control conditions during the early stages of ischaemia, as we have previously reported with first generation adenosine sensors placed on the surface of the slice (Dale *et al.* 2000). For example, after 5 min of ischaemia in nominally Ca^2+^-free medium the ado/ino levels were approximately threefold the amount measured at the comparable time point in standard aCSF (Table 1). Re-oxygenation resulted in a characteristic surge of adenosine release, but this was reduced in the Ca^2+^-free aCSF (Fig. 3a, Table 1). Overall, the total amount of adenosine released (as measured by the integral over the ischaemic and 30 min post-ischaemic period) was not different in nominally Ca^2+^-free aCSF (Table 1). This suggests that the PPE was smaller under these conditions because the enhanced early phases of adenosine release may have depleted the releasable stores of adenosine (Pearson *et al.* 2001, 2006; Frenguelli *et al.* 2003). ATP release was no different in nominally Ca^2+^-free aCSF compared to controls (Fig. 3a, Table 1). Re-introduction of Ca^2+^ to the aCSF caused the return of the fEPSP (112.6 ± 17.6% of baseline; *n* = 4). Post-ischaemic recovery of the fEPSP was greatly improved in slices exposed to ischaemia in nominally Ca^2+^-free conditions, compared to controls (7.6 ± 1.1% of baseline; *n* = 13), despite the fact that since the anoxic de-polarisation occurred earlier, the period of ischaemia extended for longer after its occurrence. This indicates full functional recovery of the hippocampal circuitry and suggests that the ATP release observed under these conditions is unlikely to be the result of catastrophic and irreversible neuronal death, at least over the time-course of these acute electrophysiological experiments.

Simple substitution of Mg^2+^ for Ca^2+^ will not remove all extracellular Ca^2+^ and processes requiring only small amounts of Ca^2+^ may still occur. We therefore omitted Ca^2+^ from the perfusion medium (substituted with an additional 2 mmol/L Mg^2+^ as before) and added 1 mmol/L EGTA to chelate any residual extracellular Ca^2+^. This more radical treatment also speeded the occurrence of the anoxic depolarisation (326 ± 24 s after the onset of ischaemia; *n* = 6). Perfusion of Ca^2+^-free/EGTA ischaemic aCSF once again caused a greater increase in adenosine release during the early phases of ischaemia compared with the control (Fig. 3b, Table 1). However, the integral of adenosine release (during and for 30 min after ischaemia) was no different from the control indicating that the high levels of adenosine release in Ca^2+^-free/EGTA ischaemic aCSF was not sustained, but instead showed some form of depletion.

In contrast to the facilitation of adenosine release by removal of extracellular Ca^2+^, ischaemic ATP release was essentially eliminated (Fig. 3b and Table 1). ATP and adenosine release thus have differing sensitivities to extracellular Ca^2+^: whereas ATP release depends upon the presence of extracellular Ca^2+^, this acts to inhibit adenosine release. The simplest explanation for the differential effects of extracellular Ca^2+^ on the release of adenosine and ATP is that the release of these two purines are independent processes and that ATP release does not substantially contribute (through extracellular degradation) to the ado/ino signal recorded during and following ischaemia.

### ATP release is not sensitive to TTX

**Fig. 4:**
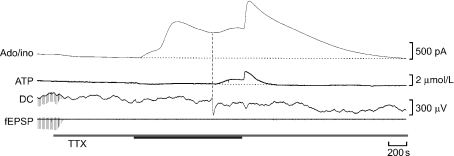
Adenosine and ATP release during ischaemia persists in presence of TTX. TTX applied at 1 μmol/L rapidly blocks synaptic transmission (and in this case causes a fall in the Ado/ino tone). Although the AD is greatly delayed (vertical dashed line) ATP release still occurs following this event. Adenosine release is unaffected by the presence of TTX. Black bar denotes period of ischaemia.

As ATP release during ischaemia depends upon the presence of extracellular Ca^2+^, we tested whether it might also depend upon neuronal spiking by applying the Na^+^-channel blocker, TTX at 1 μmol/L. This concentration of TTX was sufficient to eliminate the presynaptic fibre volley and hence synaptic transmission. It also took far longer for the slice to pass through the anoxic depolarisation (851 ± 38 s after the onset of ischaemia). However, release of ATP following the anoxic depolarisation with an unchanged magnitude was still observed (Table 1, Fig. 4). Similarly, adenosine release was unaffected by the presence of TTX. This further underlines our observation that ATP release, although Ca^2+^-dependent, appears to occur via a mechanism that is distinct from conventional synaptic release (which would also be TTX-sensitive).

### ATP release is not blocked by glutamate receptor antagonism

**Fig. 5:**
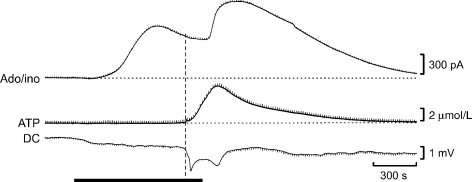
ATP and adenosine release display different sensitivities to blockade of ionotropic glutamate receptors. In the presence of 5 mmol/L kynurenic acid, the fEPSP was abolished and the anoxic depolarisation considerably delayed (dashed vertical line). The amount of adenosine release during and following ischaemia (black bar) was somewhat reduced, whereas ATP release was greatly enhanced.

We used the broad spectrum ionotropic glutamate receptor antagonist kynurenic acid (5 mmol/L; Fig. 5) to abolish excitatory synaptic transmission and determine the consequences for ischaemic purine release. In keeping with previous observations (Pearson *et al.* 2006), glutamate receptor antagonism reduced the amount of adenosine by around 25% during the early stages of ischaemia (Table 1). As the duration of the ischaemic episode (and hence the period of adenosine release) was greatly prolonged to ensure the occurrence of the anoxic depolarisation (see below), the total amount of adenosine released (the integral) was indistinguishable from the control (Table 1). These data suggest that the rate of adenosine production is reduced by ionotropic glutamate receptor blockade, but that glutamate receptor activation is not an obligatory requirement for adenosine release.

Kynurenic acid greatly delayed the anoxic depolarisation (775 ± 56 s after onset of ischaemia). Despite this increased delay, the release of ATP (*n* = 6) still followed the occurrence of the anoxic depolarisation (Fig. 5). Strangely, ATP was released at greatly enhanced levels (Table 1). This dissociation between the effects of glutamate receptor blockade on adenosine and ATP release suggests that ATP release does not contribute appreciably to the accumulation of extracellular adenosine.

### Adenosine and ATP are not released via gap junction hemichannels

**Fig. 6:**
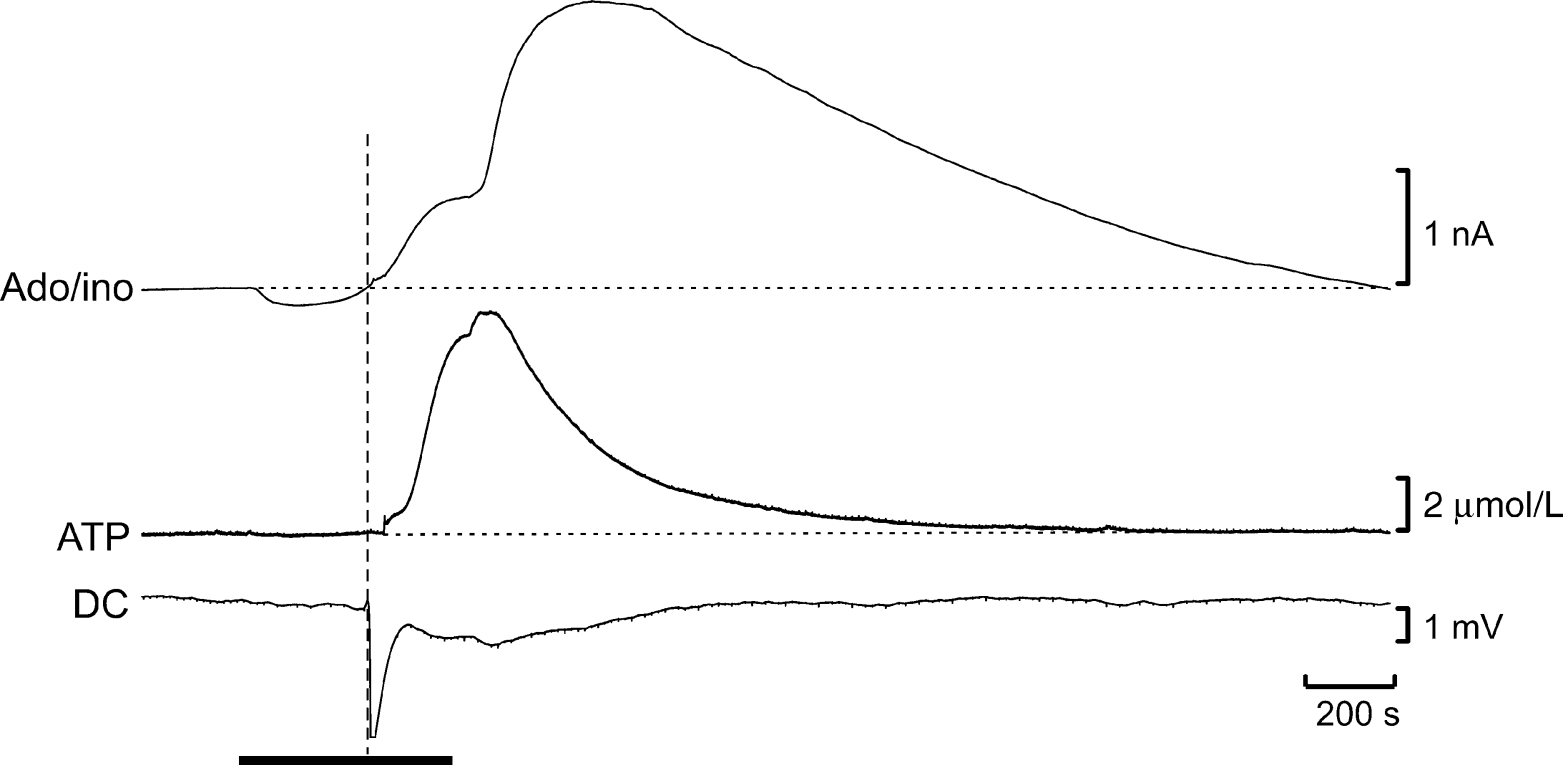
Gap junction hemichannels do not appear to mediate ischaemic purine release. The hemichannel blocker carbenoxolone (CBX, 100 μmol/L) depressed the fEPSP, but did not prevent occurrence of the anoxic depolarisation (dashed vertical line). Adenosine release was unaltered by CBX. However, ATP release was greatly increased. Black bar denotes period of ischaemia.

Both connexin and pannexin hemichannels can conduct ATP (Bao *et al.* 2004; Theis *et al.* 2005) and, in the case of connexins, have been implicated in ischaemic cell death (Contreras *et al.* 2004; Thompson *et al.* 2006). To test whether these channels could allow efflux of ATP or adenosine during ischaemic conditions, we applied carbenoxolone (CBX, 100 μmol/L; *n* = 5) in control aCSF for at least 30 min prior to an ischaemic episode. CBX caused a gradual inhibition of the fEPSP that stabilised at 22.9 ± 4.3% of baseline (data not shown). CBX had little effect on ischaemic adenosine release (Table 1), however, ATP release was greatly enhanced (Fig. 6, Table 1). By general consensus, CBX can act at many targets but the inability of CBX to inhibit either ATP or adenosine release effectively rules out a contribution of hemichannels to the release of purines during ischaemia.

### Adenosine does not arise from breakdown of ATP in the extracellular space

The presence of ectonucleotidases that can metabolise ATP to ADP, AMP and adenosine in the hippocampus is well known. These enzymes could conceivably play a role in the production of adenosine during ischaemia. We therefore tested the efficacy of the endogenous enzymes and their susceptibility to the ecto-ATPase blocker ARL 67156.

**Fig. 7:**
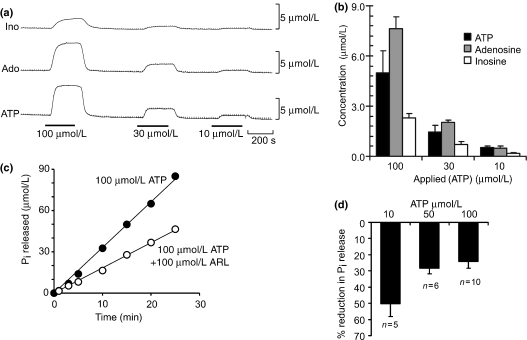
Rapid but incomplete conversion of ATP to adenosine in the extracellular space of the hippocampus. (a) Bath application of 100, 30 and 10 μmol/L ATP (indicated by black bars) was rapidly detected as adenosine and inosine by biosensors within the slice. (b) About 10% of the applied ATP was converted to adenosine or inosine. However, only around 5% of the applied ATP can be detected within the slice suggesting breakdown and accumulation of ADP/AMP. (c) Measurement, via the malachite green assay, of the time-dependent breakdown of ATP through accumulation of inorganic phosphate (Pi) in a hippocampal slice. Application of ARL 67156 substantially lowered the rate of ATP breakdown but did not abolish it. (d) Summary graph demonstrating that ARL 67156 acts as a competitive inhibitor of ATP breakdown and becomes less effective as the concentration of ATP rises.

To demonstrate the presence of endogenous ectonucleotidases activity we inserted ATP, Ado/ino and Ino biosensors into the slice to record the signals during the bath application of ATP (10–100 μmol/L). We found that about 7% of the applied ATP was converted to adenosine with a further 2–3% appearing as inosine (Fig. 7a). This conversion, although very incomplete compared with the amounts of applied ATP, nevertheless occurred very rapidly. Interestingly, in the slice the ATP biosensor only detected around 5% of the bath applied ATP, suggesting that as much as 80% of the ATP is broken down and presumably accumulates within the slice as ADP and AMP.

We made use of a convenient phosphate assay to measure the catabolism of ATP by the endogenous ectonucleotidases in the hippocampal slice to test the efficacy of ARL 67156 as a blocker. At 100 μmol/L, ARL 67156 mediated partial competitive blockade of the conversion of ATP, giving a block of some 50% against 10 μmol/L ATP and some 24% against 100 μmol/L ATP (Figs. 7c and d). Thus, if adenosine production were to arise from prior ATP release we would expect ARL 67156 to cause a partial reduction of adenosine accumulation during ischaemia.

We therefore performed ischaemia in the presence of ARL 67156 (100 μmol/L) in slices that had been pre-incubated with the blocker for at least 15 min. Interestingly, ARL 67156 caused a reduction of the adenosine tone of 110 ± 49 nmol/L (*n* = 7), suggesting that the basal levels of adenosine in the slice arise from the breakdown of ATP. This confirms the observation made in rat by Dulla *et al.* (2005) with MK I adenosine sensors, and supports the conclusions of Pascual *et al.* (2005) who suggested that release of ATP from glial cells gives rise to an adenosine tone in mouse hippocampus.

**Fig. 8:**
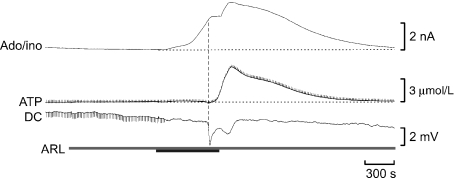
Adenosine release during ischaemia does not result from the breakdown of ATP. Application of 100 μmol/L ARL 67156 enhanced ATP release during ischaemia (black bar) but had no effect on adenosine release.

ARL 67156 caused a modest enhancement of ATP release following the anoxic depolarisation (Table 1, Fig. 8), however, it did not reveal any early phase of ATP release during ischaemia that could conceivably have been hidden from the ATP biosensor under control conditions by virtue of efficient or localised catabolism by the ectonucleotidases. Furthermore, adenosine release during ischaemia was not significantly different from the control in the presence of ARL 67156 (Table 1, Fig. 8). We therefore conclude that ATP breakdown (although it can occur and does contribute to a basal tone of adenosine) does not significantly contribute to the production of extracellular adenosine during ischaemia.

### An intracellular origin for adenosine during ischaemia

As adenosine does not appear to arise from the extracellular breakdown of ATP, it may well be released directly during ischaemia. Pre-treatment with the membrane permeable homocysteine thiolactone is an elegant method that has been used previously to ‘trap’ intracellular adenosine in the form of *S*-adenosyl homocysteate (Lloyd *et al.* 1993) through the actions of the intracellular enzyme *S*-adenosyl homocysteine hydrolase. We therefore tried the same method to test whether we could diminish adenosine release during ischaemia.

Homocysteine thiolactone is a small highly reactive molecule that is electroactive. However, limiting the concentration of homocysteine thiolactone to 100 μmol/L ensured that an extra background current of only a few hundred pA was seen on the sensors (without the screening layer even this concentration would have given a current of several 10s of nA). Pre-treatment with homocyseine thiolactone reduced the adenosine release at all phases during ischaemia. This was most marked during the PPE (Table 1). Strangely, homocysteine thiolactone enhanced ATP release (Table 1).

### Equilibrative nucleoside transporters do not mediate ischaemic purine release

The simplest explanation for our data is that adenosine is mainly released as adenosine, rather than an upstream metabolite, and that ATP and adenosine release during and following ischaemia are essentially independent processes. We therefore tested whether the application of adenosine equilibrative transport inhibitors could reduce the release of adenosine during ischaemia.

**Fig. 9:**
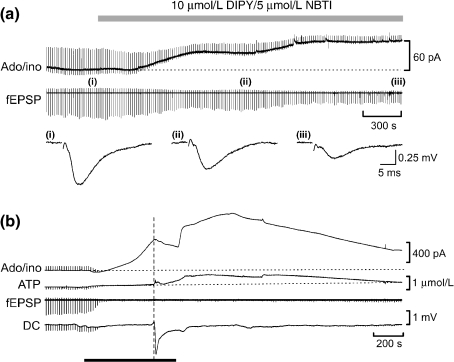
Inhibition of nucleoside transport enhances the extracellular accumulation of adenosine during ischaemia. (a) Combined application of the transport inhibitors dipyridamole (DIPY, 10 μmol/L) and NBTI (5 μmol/L) resulted in a slowly developing inhibition of the fEPSP which coincided with a slowly increasing signal on the Ado/ino sensor, demonstrating that the transport inhibitors were effective in raising extracellular adenosine levels. Magnified examples of the fEPSP are shown taken before (i) and during (ii and iii) the application of NBTI/DIPY at the times indicated. (b) Prolonged incubation with DIPY/NBTI did not prevent release of adenosine during ischaemia (black bar). Instead ado/ino release was enhanced, whilst there was little apparent effect on ATP release. The equilibrative nucleoside transporters are thus highly unlikely to mediate the release of adenosine during ischaemia.

Combined application of the equilibrative nucleoside transport (ENT) inhibitors 10 μmol/L DIPY and 5 μmol/L NBTI (*n* = 4; Fig. 6), caused a slow ∼50% depression of the fEPSP over the course of around 40 min. We and others have previously shown that this depression is the result of an increased tone of extracellular adenosine as a result of adenosine transport inhibition (Dunwiddie and Diao 1994; Pearson *et al.* 2001; Gadalla *et al.* 2004). We directly observed this gradual increase in adenosine tone in 2/3 cases with the ado/ino biosensor (Fig. 9a). Once the depression of the fEPSP had stabilised, we made the slice ischaemic to observe the effect of these ENT inhibitors on both ATP and ado/ino release (Fig. 9b). We observed greatly enhanced accumulation of adenosine during all phases of release. Overall there was a twofold increase of adenosine release compared with the control (Fig. 9b, Table 1). As might be expected the adenosine transport inhibitors had no significant effect on ATP release (Fig. 9b, Table 1).

## Discussion

Our results demonstrate that both ATP and adenosine are released during ischaemic episodes. However, the timing, characteristics and mechanisms of ATP and adenosine release differ dramatically. We shall discuss each in turn, along with the potential significance of purine release during ischaemia.

### The timing and phases of ATP and adenosine release

Whereas adenosine release occurs almost immediately following the onset of hypoxia/ischaemia (Dale *et al.* 2000; Frenguelli *et al.* 2003; Pearson and Frenguelli 2004; Pearson *et al.* 2006), ATP release only occurred after the anoxic depolarisation. The timing of ATP release remained constant relative to the anoxic depolarisation following treatments that either speeded (EGTA/zero Ca^2+^) or slowed (kynurenic acid/TTX) its occurrence. Similarly, the time to the anoxic depolarisation is longer in slices from younger animals, yet ATP release still follows this event. ATP release is thus contingent upon the events that precipitate the anoxic depolarisation.

We observed two phases of adenosine release: release during the ischaemic episode followed by a surge in release on re-oxygenation (the PPE). This is remarkably similar to the release of adenosine during hypoxia (Frenguelli *et al.* 2003). During the PPE, adenosine levels could take 15–20 min to return to the pre-ischaemic baseline and could underlie the protracted post-ischaemic adenosine-dependent depression of synaptic transmission observed by Pearson *et al.* (2006). There were also two phases of ATP release – immediate progressive release following the anoxic depolarisation, and a surge of release following re-oxygenation. By contrast to the adenosine PPE, this post-ischaemic ATP release was short-lived, and ATP levels returned to baseline within around 5 min following re-oxygenation. In contrast to the adenosine PPE, this secondary phase of ATP release also depended upon the occurrence of the anoxic depolarisation – it was not seen on re-oxygenation following episodes of ischaemia that were too short to evoke the anoxic depolarisation.

### The characteristics of ATP and adenosine release

Compared with ATP, much greater quantities of adenosine (some 17-fold) were produced during and following an ischaemic episode. Whereas adenosine could reach a maximum level of 20–30 μmol/L, peak ATP levels were restricted to 1–2 μmol/L under control conditions. Nevertheless, these levels are sufficient to activate P2 receptors, and are greatly in excess of the nanomolar levels reported in a recent microdialysis study (Melani *et al.* 2005), which may have been unable to resolve ATP release around the anoxic depolarisation.

ATP and adenosine release exhibited different sensitivities to extracellular Ca^2+^. The early phases of ischaemic adenosine release appear to be negatively regulated by extracellular Ca^2+^– its removal enhanced release – as we initially reported during hypoxia with MK I sensors (Dale *et al.* 2000) and as has been confirmed recently in cultured astrocytes (Martin *et al.* 2007). However the post-ischaemic release (the PPE) was diminished when extracellular Ca^2+^ was omitted or chelated with EGTA. This may result from depletion of the releasable pool of adenosine by enhanced early release. Importantly, during prolonged ischaemia *in vivo*, initially high levels of extracellular adenosine gradually return to baseline (Matsumoto *et al.* 1992). Thus, any treatment designed to enhance adenosine release, without also ensuring greater availability, may be of limited neuroprotective value as it would deplete the adenosine stores more quickly and lead to lowered neuroprotection during later stages of the insult.

By contrast, ATP release required extracellular Ca^2+^: release following the anoxic depolarisation was virtually eliminated by Ca^2+^ chelation with EGTA. Interestingly ATP release remained unaffected by simple removal of extracellular Ca^2+^ or the presence of TTX. As both of these manipulations will block conventional synaptic transmission, ATP release, although Ca^2+^-dependent, may differ mechanistically from conventional synaptic vesicular release.

ATP and adenosine release were distinguished further by their dependence on glutamate receptors. Kynurenic acid reduced adenosine release early in the ischaemic episode, consistent with the partial NMDA receptor-dependence of adenosine release during ischaemia (Pearson *et al.* 2006). By contrast ATP release was greatly enhanced by kynurenic acid. Interestingly, glutamate release is known to occur after the anoxic depolarisation (Rossi *et al.* 2000) and could therefore limit the subsequent amount of ATP release.

### Does ATP breakdown contribute to adenosine production during ischaemia?

A key issue is whether extracellular adenosine during ischaemia results from direct adenosine release or the prior release of ATP and its subsequent conversion to adenosine via the actions of the ectonucleotidases. Previous work from our laboratory, on the basis of pharmacological inhibition of cAMP transport and ecto-5′-nucleotidase, and their minimal effect on the hypoxic depression of excitatory synaptic transmission, argued against the likelihood of released adenine nucleotides as contributors to extracellular adenosine during metabolic stress (Pearson *et al.* 2003; Pearson and Frenguelli 2004).

Our new data directly suggest adenosine release that is largely independent from the release of ATP. Firstly, adenosine release occurred in the apparent absence of any ATP release; secondly, ATP release and adenosine release exhibited differential sensitivities to both extracellular Ca^2+^ and glutamate receptor blockade; thirdly, ATP release was only observed following the anoxic depolarisation, whereas adenosine release exhibited no such dependence on this event. Any hypothesis that maintained production of adenosine from prior release of ATP would have to be consistent with these apparent dissociations of mechanism between the two processes and would thus be have to be highly complex.

We have also directly addressed this issue in two further ways: by examining ATP breakdown in the slice; and by testing whether inhibition of the ectoATPases can alter ATP and adenosine release during ischaemia.

As might be expected, ATP conversion to adenosine does occur in the slice. Although it is rapid, only about 10% of exogenously applied ATP is converted to adenosine or downstream purines. This contrasts with the report of Dunwiddie and colleagues (Dunwiddie *et al.* 1997) who indirectly monitored conversion of ATP to adenosine in hippocampal slices, through adenosine A_1_ receptor-mediated activation of a K^+^ conductance, and who suggested complete conversion of ATP to adenosine. The simplest interpretation of our observation is that in order to see a signal of, for example, 3 μmol/L adenosine at 5 min of ischaemia (cf. Table 1) around 30 μmol/L ATP would have to be released – no ATP is observed at this time point. As the lower detection limit of the ATP biosensors is below 100 nmol/L it is very likely that we would detect this hypothesised ATP release if it were to occur. Similarly, if the adenosine PPE was entirely the result of the breakdown of ATP we would predict release at a level in excess of 200 μmol/L to give rise to the observed adenosine signal (∼23 μmol/L) – however, only 1.8 μmol/L is observed (cf. Table 1).

Although the inhibitors of ectonucleotidases are neither particularly potent nor specific we have confirmed that ARL 67156 has some useful inhibitory activity against the ectoATPases in hippocampus (Figs. 7c and d). Although ARL 67156 reduced the adenosine tone by some 100 nmol/L and enhanced ATP release during ischaemia, it did not reveal any hypothesised early phase of ATP release that could give rise to the early phases of adenosine release during ischaemia. Even more importantly ARL 67156 had no significant effect on adenosine production during ischaemia. We therefore conclude that while ATP conversion to adenosine in the hippocampus undoubtedly occurs, this mechanism is unlikely to make a significant contribution to the large accumulation of extracellular adenosine during ischaemia

### Mechanisms of ATP and adenosine release

The ATP release that we observed is unlikely to result from the breakdown of cellular membranes and cytolysis: we could evoke the anoxic depolarisation and ATP release during conditions (most notably Ca^2+^-free aCSF, where it was directly tested) that permitted full recovery of excitatory synaptic transmission – an observation inconsistent with irreversible cellular damage.

Accordingly, there are other possible routes for ATP and adenosine to cross the plasma membrane during and after the ischaemic episode: via exocytosis; through a transporter; or via an open channel (Volterra and Meldolesi 2005; Franke and Illes 2006). That adenosine release persisted in the absence of extracellular Ca^2+^ and TTX strongly militates against conventional exocytotic release. However, as ATP release did exhibit Ca^2+^-dependence (despite being TTX-independent), an exocytotic release mechanism during ischaemia remains possible. Glial cells release ATP in the hippocampus via a SNARE-dependent process (Pascual *et al.* 2005), which is converted to adenosine and contributes to a tone of adenosine in the mouse hippocampus. Our data also directly demonstrate that ATP breakdown contributes to an adenosine tone in rat and supports a similar mechanism for its origin – release from glial cells.

We also tested whether gap junction hemichannels might contribute to purine release by applying CBX. That adenosine and ATP release persisted (and indeed ATP release was greatly enhanced by this treatment) strongly suggests that gap junction hemichannels cannot be responsible for the release of these molecules during ischaemia (see also Martin *et al.* 2007), despite the recent observation that these channels open under these conditions (Thompson *et al.* 2006). We cannot, however, eliminate the possibility that some other large conductance channel activated by the anoxic depolarisation could contribute to their release, although P2X_7_ pores have recently been excluded as conduits of adenosine release during hypoxia (Martin *et al.* 2007).

The equilibrative adenosine transporters, in particular ENT1 and ENT2, are important regulators of extracellular adenosine (King *et al.* 2006). We and others have found that combined application of the transport blockers dipyridamole and NBTI increased the extracellular adenosine tone, measured directly in this study, with significant A_1_R-dependent effects on excitatory synaptic transmission (Dunwiddie and Diao 1994; Pearson *et al.* 2001; Gadalla *et al.* 2004). This implies a constant cycling of adenosine between the extra- and intracellular compartments and it has been suggested that specific cell types could contribute differentially to the release and removal of adenosine from the extracellular space (cf. Fredholm *et al.* 1994). Our observation that adenosine transport blockers enhanced rather than diminished release of adenosine during ischaemia is consistent with the suggestion that equilibrative transporters may limit its accumulation in the extracellular space (Cunha 2005) and with observations *in vivo* (Phillis *et al.* 1989). However the precise molecular and cellular mechanism of adenosine release during ischaemia remains unresolved, although recent evidence implicates astrocytes as the primary source of adenosine during hypoxia (Martin *et al.* 2007). Nonetheless, as adenosine transport inhibitors are known to be neuroprotective (Noji *et al.* 2004), their efficacy likely results from their ability to dramatically increase levels of extracellular adenosine during all phases of ischaemia and post-ischaemic recovery.

During ischaemia, glutamate is released via reversed Na^+^-dependent transport (Jabaudon *et al.* 2000; Rossi *et al.* 2000). This occurs following the anoxic depolarisation as neurons become unable to maintain the physiological Na^+^ gradients across their membranes (Somjen 2001). This reversal of the Na^+^-gradient could in principle power an adenosine efflux via the Na^+^-dependent concentrative transporters (Gray *et al.* 2004). However, adenosine release occurs almost immediately following the onset of ischaemia and presumably well in advance of loss of the normal transmembrane Na^+^ gradients. Thus, although Na^+^-dependent efflux of adenosine could contribute to later stages of adenosine release, some other as yet unknown mechanism must underlie its release during the earlier stages of ischaemia.

### The significance of tonic and evoked ATP and adenosine release

Numerous estimates of basal extracellular adenosine concentration have set the value at between 30 and 300 nmol/L (Fredholm *et al.* 2001). This would provide tonic activation of at least the high affinity and high abundance A_1_ receptor. That such a tone exists is amply documented by the many studies showing enhancements of hippocampal synaptic transmission by A_1_R antagonists (Bauman *et al.* 1992; Dulla *et al.* 2005; Pascual *et al.* 2005; Studer *et al.* 2006) or by adenosine deaminase (Gadalla *et al.* 2004). The extent of the tone and subsequent enhancement by antagonists may be determined by the particular experimental setup (Moore *et al.* 2003; Kukley *et al.* 2005). Use of ARL 67156 in our study suggests a basal tone of adenosine of approximately 100 nmol/L. Increases in basal tone could be detected (with consequent depression of synaptic transmission) in the presence of adenosine uptake blockers (Fig. 9). However, 30 min after the end of ischaemia, adenosine levels were still elevated, a finding consistent with our previous observations (Pearson *et al.* 2006).

*In vivo*, basal ATP levels have been estimated to be as low as a few nmol/L (Phillis and O’Regan 2003; Melani *et al.* 2005), which is below the level of detection in the present study. Accordingly, it is not clear whether this low basal level of ATP is sufficient to exert any influence via P2 receptors (Dulla *et al.* 2005; da Silva *et al.* 2005).

Notwithstanding the vexed issue of sources of purines during metabolic stress, it is surprising that brain tissue should release ATP at a time when cellular levels of ATP are severely depleted. This ATP release may well be significant in the pathology of stroke as it is likely to deplete further the intracellular stores of ATP, delay their recovery following re-oxygenation and initiate potentially injurious signalling cascades in neighbouring tissue. This is especially pertinent in the context of human anoxic depolarisation-like events (peri-infarct depolarisations) which spread through the penumbra enlarging the area of damaged brain tissue (Fabricius *et al.* 2006).

Accordingly, P2 receptor antagonists are neuroprotective in a range of models (Franke and Illes 2006) implying that ATP release and subsequent activation of P2 receptors, which may occur for some time after the end of ischaemia, may be harmful to cells. Post-ischaemic up-regulation of the ectonucleotidases responsible for metabolism of extracellular ATP has been described (Braun *et al.* 1998) which may represent an adaptive mechanism to reduce the damaging effects of ATP release in further ischaemic attacks. In contrast, proliferation of astrocytes or migration of microglia in response to ATP may provide trophic support for injured tissue (Franke and Illes 2006).

Adenosine release has been reported to be neuroprotective through its action on A_1_ receptors, but damaging through A_2A_ receptors (Cunha 2005). Our data demonstrate that adenosine release is maintained well after the ischaemic insult has terminated and indeed involves a surge of release following re-oxygenation (the PPE). The significance of the PPE remains an intriguing mystery as we have yet to find a means to manipulate it selectively. Nevertheless, a PPE-like mechanism *in vivo* could be of significance around the boundary of an infarction. In this peripheral zone the enhanced adenosine release could assist reperfusion by acting as a vasodilatator (Meno *et al.* 1993) and limit the spread of glutamatergic excitation (Pearson *et al.* 2006).

In conclusion, the rapid ischaemic release of adenosine and the subsequent release of ATP during the anoxic depolarisation are governed by distinct temporal and mechanistic processes. The implications of purine release for post-ischaemic neuronal function are likely therefore to depend upon the balance between beneficial and damaging cellular cascades initiated by the two purines. Understanding how to tip the balance in favour of benefit might be useful in the treatment of disorders of the human CNS.

## Abbreviations used:

aCSF: artificial cerebrospinal fluid
CBX: carbenoxolone
DIPY: dipyridamole
ENT: equilibrative nucleoside transporter
fEPSP: field excitatory postsynaptic potentials
NBTI: S-(4-Nitrobenzyl)-6-thioinosine
PPE: post-hypoxic/ischaemic purine efflux
